# Unilateral parotitis following mRNA coronavirus disease 2019 vaccination

**DOI:** 10.1590/0037-8682-0059-2022

**Published:** 2022-04-29

**Authors:** Tumay Bekci, Ismet Mirac Cakir, Serdar Aslan

**Affiliations:** 1 Giresun University, Faculty of Medicine, Department of Radiology, Giresun, Turkey.

**FIGURE 1:**
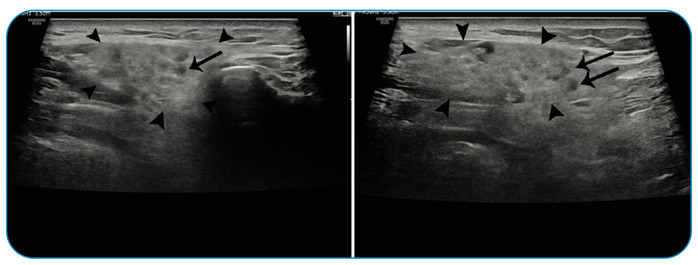
Sonographic examination showed increased echogenicity and thickness of the right parotid gland **(arrowheads)**. Hypoechoic areas **(arrows)** were noted within the gland, which were consistent with the features of parotitis.

A 69-year-old male patient was admitted to our hospital due to complaints of right-sided facial swelling and pain, especially in the pre-auricular area and cheek, that lasted for 3 days. Upon physical examination, non-fluctuant facial swelling was observed, but no purulent discharge was noted after parotid massage. The patient had no other symptoms. All standard laboratory examinations were performed. The patient tested negative for mumps IgM and IgG antibodies. Based on the patient’s detailed history, he received the third dose of mRNA coronavirus disease 2019 COVID-19 vaccine 10 days prior to admission. The patient did not use any medication and had no history of autoimmune disease. Sonographic examination showed increased echogenicity and thickness of the right parotid gland. Hypoechoic areas were noted within the gland, which were consistent with the features of parotitis (Figure 1, arrow). The patient was suspected of having parotitis possibly associated with the mRNA COVID-19 vaccination. The patient was monitored, but no medication treatment was provided; his symptoms eventually resolved 3 days later. Hence, acute parotitis associated with the mRNA COVID-19 vaccination was reported in adults^1^ ^,^ ^2^. The underlying mechanism is likely a cross-reaction between the coronavirus spike protein targeted with an mRNA vaccine and parotid cell antigens. A few cases of thyroiditis after mRNA COVID-19 vaccination with a similar mechanism have been reported in the medical literature^3^. However, parotitis occurring after an mRNA COVID-19 vaccination has not been reported. Hence, radiologists and clinicians should be aware of this rare condition.
